# In Silico Analysis Reveals High Levels of Genetic Diversity of *Plasmodium knowlesi* Cell Traversal Protein for Ookinetes and Sporozoites (*PkCelTOS*) in Clinical Samples

**DOI:** 10.3390/tropicalmed8080380

**Published:** 2023-07-26

**Authors:** Md Atique Ahmed, Pratisthita Baruah, Ahmed Saif, Jin-Hee Han, Mohammed Al-Zharani, Syeda Wasfeea Wazid, Saad Alkahtani, Saurav J. Patgiri, Mohammed S. Al-Eissa, Fu-Shi Quan

**Affiliations:** 1 ICMR-Regional Medical Research Centre, NE Region, Dibrugarh 786010, Assam, Indiasaurav.patgiri@gmail.com (S.J.P.); 2 Department of Clinical Laboratory Sciences, Faculty of Applied Medical Sciences, King Khalid University, Abha 61321, Saudi Arabia; 3 Department of Medical Environmental Biology and Tropical Medicine, School of Medicine, Kangwon National University, Chuncheon 24341, Republic of Korea; 4 Biology Department, College of Science, Imam Mohammad Ibn Saud Islamic University (IMSU), Riyadh 11623, Saudi Arabiamsaleissa@imamu.edu.sa (M.S.A.-E.); 5 Arogyo Society of Health, Welfare and Support (ASHWAS), Guwahati 785640, Assam, India; wasfeea@gmail.com; 6 Department of Zoology, College of Science, King Saud University, Riyadh 11451, Saudi Arabia; 7 Medical Research Center for Bioreaction to Reactive Oxygen Species and Biomedical Science Institute, Core Research Institute (CRI), Kyung Hee University, Seoul 02447, Republic of Korea; 8 Department of Medical Zoology, School of Medicine, Kyung Hee University, Seoul 02447, Republic of Korea

**Keywords:** *Plasmodium knowlesi*, CelTOS, genetic diversity, natural selection

## Abstract

The cell-traversal protein for ookinetes and sporozoites (CelTOS), expressed on the surface of ookinetes and sporozoitesin *Plasmodium species*, is a promising malaria vaccine candidate. CelTOS is essential for parasite invasion into mosquito midgut and human hepatocytes, thereby contributing to malaria transmission and disease pathogenesis. This study explores the genetic diversity, polymorphisms, haplotypes, natural selection, phylogenetic analysis, and epitope prediction in the full-length *Plasmodium knowlesi CelTOS* gene in clinical samples from Sarawak, Malaysian Borneo, and long-term laboratory strains from Peninsular Malaysia and the Philippines. Our analysis revealed a high level of genetic variation in the *PkCelTOS* gene, with a nucleotide diversity of π ~ 0.021, which was skewed towards the 3’ end of the gene. This level of diversity is double that observed in *PfCelTOS* and 20 times that observed in *PvCelTOS* from worldwide clinical samples. Tests of natural selection revealed evidence for positive selection within clinical samples. Phylogenetic analysis of the amino acid sequence of *PkCelTOS* revealed the presence of two distinct groups, although no geographical clustering was observed. Epitope prediction analysis identified two potential epitopes (96AQLKATA102 and 124TIKPPRIKED133) using the IEDB server and one epitope (125IKPPRIKED133) by Bcepred server on the C’ terminal region of *PkCelTOS* protein. Both the servers predicted a common epitope region of nine amino acid length (IKPPRIKED) peptide, which can be studied in the future as a potential candidate for vaccine development. These findings shed light on the genetic diversity, polymorphism, haplotypes, and natural selection within *PkCelTOS* in clinical samples and provide insights about its future prospects as a potential candidate for *P. knowlesi* malaria vaccine development.

## 1. Introduction

Malaria is a life-threatening disease predominant in most tropical and sub-tropical countries. The disease is transmitted when a female *Anopheles* mosquito is infected with *Plasmodium* spp. and injects the parasite into the human bloodstream [1]. The illness is of significant concern to public health and the well-being of humankind. The World Malaria Report 2022, published by the World Health Organization (WHO), revealed that approximately 247 million malaria cases occurred in 84 countries where malaria is endemic. The initial year of the SARS-CoV-2 pandemic (2019–2020) saw the most significant annual increase in malaria cases, believed to be caused by the pandemic-related disruptions during this time [2]. Out of the different *Plasmodium* species, nine have been identified to cause infection in humans. *Plasmodium vivax*, *P. falciparum*, *P. ovale curtisi*, *P. ovale wallikeri*, and *P. malariae* are human malaria parasites well-known to cause malaria in humans [1,3,4,5]. In addition, some *Plasmodium* parasites of simian origin, e.g., *P. knowlesi*, *P. simium*, *P. brasilianum*, and *P. cynomolgi,* have also been reported to infect humans [4,6,7]. Among the parasites infecting humans, the most significant threat comes from *P. falciparum* and *P. vivax*. *P. falciparum* exhibits the highest virulence and is responsible for the highest mortality, especially in the African continent [8]. In many countries outside of sub-Saharan Africa, *P. vivax* is the most prevalent species [1]. The simian malaria parasite *P. knowlesi* has been discovered to cause human infection, as reported from several Southeast Asian countries, particularly in Malaysian Borneo [4,9,10,11,12,13,14]. Due to its increasing incidence in recent years, *P. knowlesi* malaria has become a significant public health issue in the region [4,9,13,15]. The mosquito, *Anopheles hackeri,* was first identified as the vector carrying *P. knowlesi* in Malaysia [16]. Later on, many species of the Leucosphyrus group under the genus *Anopheles* were reported to transmit the parasite from macaques to humans [9,13,15]. Previous studies utilizing whole-genome and genetic analysis on clinical samples from Malaysian Borneo and Sarawak have identified the existence of at least three sub-populations [10,11,17]. Among these, two are associated with the primary monkey hosts, long-tailed macaques (*Macaca fascicularis*) and pig-tailed macaques (*Macaca nemestrina*) [15,18]. *Presbytis melalophos,* or banded leaf monkeys, are also reported to harbor *P. knowlesi* naturally [19]. *P. knowlesi* malaria can cause severe and complicated malaria cases in humans, with symptoms similar to other malaria, including fever, headache, vomiting and diarrhea, acute respiratory distress syndrome, and multi-organ failure like kidney failure [10,20]. The 24 h red blood cell life cycle of *P. knowlesi* can lead to a swift rise in parasitemia, potentially resulting in death [10]. *P. knowlesi* cannot be accurately identified through microscopy because its early trophozoites have the same features as *P. falciparum*, and its later stages resemble the band-form of trophozoites of *P. malariae* [13,21,22]. PCR is necessary as a confirmatory test [22,23]. In Malaysia, a significant increase in cases of *P. knowlesi* has been witnessed in humans over the last decade, which is presumably due to the changes in the environment like large-scale deforestation and land exploration. This might have played a key role in increasing human exposure to anopheline vectors [15,24]. Thus, there is a need for control measures such as the development of vaccines to halt transmission.

The prime challenge of developing a vaccine can be attributed to the genetic diversity found in the field samples within the potential vaccine candidates leading the vaccine to be non-efficacious [17,25]. Studies conducted on potential sporozoite-stage vaccine candidates of *P. vivax* have revealed that the sporozoite-expressed genes exhibit a higher degree of conservation than the merozoite genes [26]. The RTS,S/AS01 vaccine, under the commercial name *Mosquirix* targeting *P. falciparum* circumsporozoite protein (CSP), is the world’s first malaria vaccine to receive approval from WHO and is in current use [27,28]; however, high antigenic diversity in clinical samples have reduced the efficacy of the vaccine [25]. Thus it is important to study the antigenic diversity of potential ortholog genes of *P. falciparum* and *P. vivax* in *P. knowlesi* to develop an efficacious vaccine candidate for *knowlesi* malaria. Various studies have characterized merozoite invasion genes in *P. knowlesi,* analyzing their genetic diversity and population structure [10,11,12,17,25,29]; however, multi-stage vaccine candidates other than CSP [30] have not been genetically characterized in *P. knowlesi.*

Cell-traversal protein for ookinetes and sporozoites, briefly known as CelTOS, is a surface protein expressed in sporozoites and ookinetes in the sporogonic stages of *Plasmodium’s* life cycle. It is an essential micronemal protein that enables the parasite to cross host cell barriers [31,32,33,34]. It is necessary during parasite invasion into mosquito midgut and human hepatocytes, making it crucial for malaria transmission and disease pathogenesis [32,33,35]. A study has shown that targeted disruption of *P. berghei CelTOS* gene can significantly reduce the parasite’s ability to infect both mosquito host and human liver, almost eliminating the ability of the parasite to pass through host cells successfully [35]. Another study carried out recently discovered that CelTOS facilitates the parasite exit and completion of the cell-traversal process by rupturing the plasma membrane from inside the cells of infected human and mosquito hosts [36]. Antibodies targeting PfCelTOS have demonstrated the ability to obstruct sporozoite traversal of hepatocytes [37], and immunization of mice models with PbCelTOS provided protection against *P. berghei*, establishing its potential as a vaccine candidate [38]. Ex vivo ELISPOT studies demonstrated that peptides derived from PfCelTOS induced both proliferative and IFN-γ responses in peripheral blood mononuclear cells (PBMCs) obtained from human participants immunized with irradiated sporozoites [39]. In another study, significant inhibition of sporozoite hepatocyte infection was observed while immunizing mice with recombinant PfCelTOS in conjugation with either glucopyranosyl lipid adjuvant-liposome-QS21 (GLA-LSQ) or glucopyranosyl lipid adjuvant-stable emulsion (GLA-SE) adjuvant system [40]. Furthermore, in experimental studies conducted in vivo, it was observed that monoclonal antibodies specifically designed to target PfCelTOS showed significant inhibition of oocyst growth [40]. This effect was observed in both *P. berghei* and *P. falciparum* parasites that expressed PfCelTOS in *Anopheles gambiae* mosquitoes [40], suggesting CelTOS as a potential multi-stage vaccine candidate, as blocking this single protein can stop more than one process in the pre-erythrocytic stage [31]. A recombinant *E. coli*-expressed PfCelTOS-based vaccine is already in the Phase 1 trial [41].

Prior studies have explored *CelTOS* genetic diversity in *P. vivax* and *P. falciparum*, while none have investigated *P. knowlesiCelTOS.* Thus, in the present study, we aimed to find the genetic diversity, polymorphisms, and natural selection operating on full-length *PkCelTOS* gene sequences from 34 samples (30 clinical samples from Malaysian Borneo and 4 laboratory lines from Peninsular Malaysia and the Philippines). We also conducted a phylogenetic analysis to find the relationship between *PkCelTOS* amino acid sequences and their orthologs in other species. We predicted potential epitopes of *PkCelTOS*, which might induce an immune response during the sporozoite and ookinete stages of the parasite. Since our study is the first to examine *CelTOS* sequences from clinical samples of this zoonotic parasite, the study findings will contribute to the rational design and formulation of ookinete and sporozoite stage vaccines that are effective against *P. knowlesi.*

## 2. Materials and Methods

### 2.1. Sequence Information of PkCelTOS

A total of 34 *PkCelTOS* full-length gene (from 1 to 558 nt) sequences (4 laboratory strains including the reference H-strain PKNH_1436200 and 30 clinical samples) were obtained from the public database European Bioinformatics Institute (https://www.ebi.ac.uk/ena/browser/home, accessed on 20 March 2023) [18]. These sequences were derived from clinical samples obtained from Malaysian Borneo, as indicated in Supplementary Table S1, which shows the geographical locations of the sample collections [18]. Only high-quality, full-length *PkCelTOS* gene sequences were included for analysis. The sequences were aligned using the CLUSTAL-W program within the MegAlignLasergene v 7.0 software (DNASTAR, Madison, WI, USA). SignalP-5.0 prediction software (https://services.healthtech.dtu.dk/services/SignalP-5.0/) was used to predict the signal peptide for the full-length *PkCelTOS* protein [42].

### 2.2. Phylogenetic Analysis

Phylogenetic analysis was carried out to establish the intra-species relationship between amino acid sequences of *PkCelTOS* derived from clinical samples in Malaysian Borneo and long-term lab strains from Peninsular Malaysia and Philippines (H-strain, MR4, Philippine strain, and Malayan Strain). Phylogenetic analysis was conducted in MEGA 5.0 software utilizing Maximum Likelihood (ML) method on the basis of the Poisson correction model with 1000 bootstrap replicates [43]. Ortholog sequences of CelTOS in *P. coatneyi* (PCOAH_00055260), *P. cynomolgi* (PCYB_144300), *P. vivax* (PVX_123510), and *P. falciparum* (PF3D7_1216600) were also added to investigate the evolutionary relationship between the CelTOS protein of *P. konwlesi* along with its closest members in the genus *Plasmodium*.

### 2.3. Sequence Diversity and Polymorphism

Nucleotide diversity (π) in the *PkCelTOS* full-length gene was determined using DnaSP v6.12.03 software [44]. Other parameters, i.e., synonymous (S), non-synonymous substitutions (NS), polymorphic sites, the number of parsimony informative sites, singletons, the number of haplotypes (H), and haplotype diversity (HD) were also determined by the use of the same software. DnaSP software was utilized to visually represent nucleotide diversity by considering a window length of 50 and a step size of 10 bp.

### 2.4. Natural Selection Test

To determine the natural selection within *PkCelTOS* at the intra-species level, the neutrality tests of Tajima’s D, Fu, and Li’s F*and D* were computed using DnaSP software. Tajima’s D statistic yields a value of zero under conditions of neutrality. A negative Tajima’s D result determines population expansion, while a positive and significant Tajima’s D result signifies positive selection or balancing selection. Using the DnaSp software, the results of Tajima’s D were also shown graphically (with a window length of 50 and step size of 10 bp). Positive and significant values of Fu and Li’s F* and D* suggest that the population may have undergone a contraction. Conversely, an excess of singletons or negative values can indicate population expansion. In addition, the method of Nei and Gojobori was utilized for calculating the rate of non-synonymous substitution per non-synonymous site (dN) and the rate of synonymous substitution per synonymous site (dS) [43].

The robust McDonald and Kreitman (MK) test was carried out to examine natural selection at the inter-species level, utilizing DnaSP v6.12.03 software using 34 *PkCelTOS* gene sequences with their closely related ortholog sequences in *P. coatneyi* (PCOAH_00055260), *P. cynomolgi* (PCYB_144300) and *P. vivax* (PVX_123510).

### 2.5. Codon-Based Test Using Datamonkey Web Server for Natural Selection

Codon-based analysis of selection was performed on the *PkCelTOS* gene using the Datamonkey Web Server (https://www.datamonkey.org/ accessed on 1 July 2023) [45]. The analysis employed multiple methods, including single-likelihood ancestor counting (SLAC), mixed effects model of evolution (MEME), and fast unconstrained Bayesian approximation (FUBAR). Default settings for the significance level provided by the Datamonkey server were utilized for this analysis [45].

### 2.6. Epitope Prediction

B-cell epitopes are specific sites on a protein that can be recognized by the antigen-binding sites present on the immunoglobulin molecules. These epitopes play a crucial role in the formulation of peptide-based vaccines, disease detection, and allergy studies [46]. In this study, in silico prediction of B cell epitopes was carried out in the *PkCelTOS* sequence of *P. knowlesi*. The epitope prediction was conducted using two servers; the IEDB Analysis resource (http://tools.immuneepitope.org/bcell), with the Emini Surface Accessibility Prediction model to identify B-cell epitopes on the surface [47], and the Bcepred server (https://webs.iiitd.edu.in/raghava/bcepred/index.html) [46], using the Exposed surface method [48]. The method compares the possible conformations taken by protein side chains to energy calculation results using van der Waals interactions and steric hindrance. It assumes that the folded protein structure puts minimum strain on side-chain conformations, favoring low-energy positions during folding [48]. Since *P. knowlesi* and *P. vivax* are phylogenetically close and several studies have shown the importance of conserved cross-species vaccine candidates [11,49,50]. Therefore, in addition to identifying epitopes in *P. knowlesi,* this study examined the likelihood of epitope conservation with *P. vivax*.

## 3. Results

### 3.1. PkCelTOS Sequence Identity among Ortholog Members

**Figure 1:**
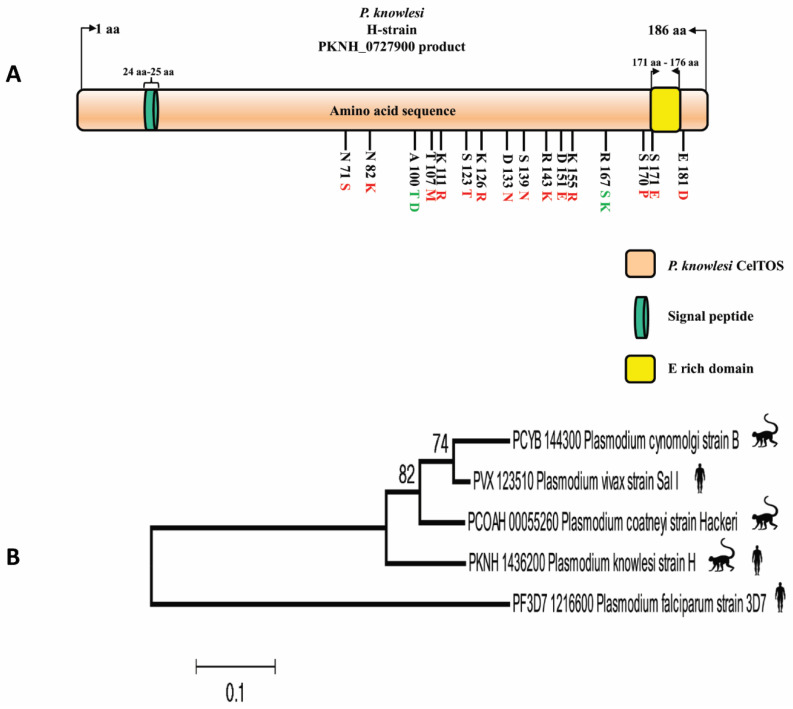
(**A**) Distribution of the amino acid polymorphism found in 34 clinical samples of the *PkCelTOS* protein. The black lines with corresponding letters and numbers indicate the amino acid positions with polymorphism. The letter preceding the number represents the amino acid found in the reference H-strain. The polymorphic variants are shown in red (two variants) and green (three variants) colors. The signal peptide region lies between amino acid positions 24 to 25 (highlighted in dark green). An E-(Glutamic acid) rich region was identified spanning the amino acid positions 171 to 176 within the alignment. “aa” refers to amino acid positions. (**B**) Phylogenetic tree showing the inter-species relationship between sequences of amino acid of CelTOS of *P. knowlesi* reference strain H with its orthologs in *P. vivax* strain Sal I, *P. coatneyi* strain Hackeri, *P. cynomolgi* strain B and *P. falciparum* strain 3D7 constructed using MEGA 5.0 software using ML method and Poisson correction model.

A signal peptide spanning from amino acid position 24 to 25 was predicted by the Signal IP server within the *PkCelTOS* protein (Supplementary Figure S1). Alignment of full-length amino acid sequences of CelTOS of *P. knowlesi* strain H with its orthologs showed that *PkCelTOS* has 84.9%, 83.8%, 78.3%, and 44.5% amino acid sequence identities with *P. viax*, *P. coatneyi*, *P. cynomolgi,* and *P. falciparum*, respectively (Supplementary Figure S2). Figure 1A depicts a schematic structure of the *PkCelTOS* protein, highlighting the observed amino acid polymorphism, which includes both two and three variants.

### 3.2. Phylogenetic Analysis of P. knowlesi CelTOS and Its Orthologs in Other Plasmodium Species

The inter-species phylogenetic relationship between *PkCelTOS* and its orthologs shows that *PkCelTOS* is phylogenetically closer to *P. vivax* than *P. coatneyi* and *P. cynomolgi*. While *P. falciparum* CelTOS is most distantly to *PkCelTOS* (Figure 1B).

**Figure 2:**
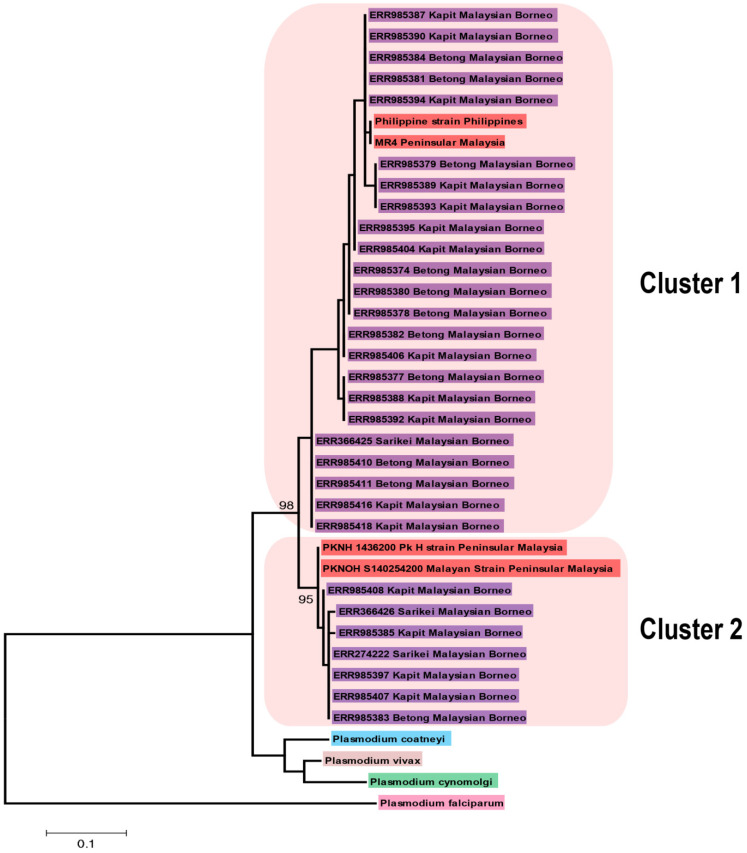
Phylogenetic tree comprising 34 *PkCelTOS* amino acid sequences (including the reference H-strain) from different regions of Malaysia and its orthologs in *P. vivax* strain Sal I, *P. coatneyi* strain Hackeri, *P. cynomolgi* strain B and *P. falciparum* strain 3D7 gene constructed using by MEGA 5.0 software using Maximum-Likelihood Model and Poisson correction model with 1000 bootstrap replicates. Bootstrap values higher than 95 were only shown in the figure. Sequences originating from Peninsular Malaysia and the Philippines are shaded in red and Malaysian Borneo in violet.

A phylogenetic analysis of 34 full-length deduced amino acid sequences of *PkCelTOS* with its orthologs utilizing the Maximum Likelihood method showed no geographical clustering; however, samples were bifurcated into two major groups supported with strong bootstrap values (Figure 2).

### 3.3. Nucleotide Diversity and Polymorphism of PkCelTOS in Clinical Samples

**Figure 3:**
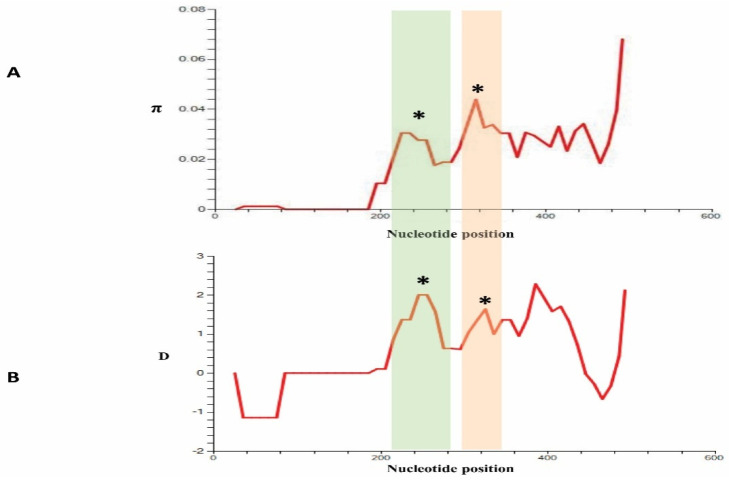
(**A**) Graphical depiction of nucleotide diversity (π) and (**B**) Tajima’s D value (D) within 34 clinical samples of the *P. knowlesi CelTOS* gene. The window length and step size for both graphs were set at 50 and 10, respectively, as implemented in DnaSP software v6.12.03. Peaks marked with an asterisk (*) on the Tajima’s D graph may indicate possible epitope regions among the clinical samples analyzed. The shaded region highlights (green and red) areas of shared high nucleotide diversity and positive Tajima’s D values, which may have possible epitopes.

Analysis of 34 *PkCelTOS* nucleotide sequences showed 28 polymorphic sites, among which 12 were synonymous substitutions, and 16 were non-synonymous. The overall nucleotide diversity of *PkCelTOS* was determined to be π = 0.02111 + 0.00105 (Table 1), which is graphically represented in Figure 3A. Out of the 24 parsimony informative sites observed in *PkCelTOS*, 5 were singleton variable sites, 22 were with two variants, and 2 were with three variants. The analysis also revealed the presence of 17 haplotypes, which exhibited a high haplotype diversity (Hd) of 0.954 ± 0.016. Figure 1A illustrates a schematic representation of the 16 non-synonymous substitutions that were observed within the 34 samples, with respect to the *P. knowlesi* reference H-strain. The nucleotide and amino acid polymorphisms of the clinical strains with respect to the reference H-strain, along with nucleotide positions, were illustrated in Supplementary Figures S3 and S4.

### 3.4. Natural Selection in PkCelTOS

Analysis of the natural selection of *PkCelTOS* gene sequences obtained from 34 sequences showed overall Tajima’s D value to be D = 1.52705, *p* > 0.10, which was not significant (Table 1). Tajima’s D values indicated again that the *PkCelTOS* gene had undergone positive selection. The Tajima’s D result is shown graphically in Figure 3B. The values of Fu and Li’s F* and D* for *PkCelTOS* were found to be positive at 0.63460 and 1.10034, respectively, but they did not attain statistical significance (Table 1). In addition, the results from the codon-based Z-test for positive selection using the Nei and Gojobori method showed that the value was found to be negative (dN-dS = −1.058, *p* > 0.05) but not significant.

The robust MK test, which takes into consideration inter-species natural selection over time, suggested that the *PkCelTOS* gene may be undergone positive selection when tested with *CelTOS* orthologs in *P. vivax*, *P. cynomolgi* and *P. coatneyi* (Table 2). Though MK test results were not statistically significant, the NI values were indicative of a positive natural selection. This may be due to the low number of sequences analyzed in this study.

### 3.5. Codon-Wise Analysis

The departure of neutrality of the *PkCelTOS* gene was assessed using codon-based tests such as FUBAR, MEME, and SLAC, which detects sites with different selection pressure. The analysis conducted using FUBAR identified five sites (82, 100, 111, 170, and 171), while MEME detected one (171) site undergoing positive selection. Analysis conducted using the SLAC method 1 (111) site detected to be under positive selection. Remarkably, both FUBAR and MEME concurred on amino acid position 171, undergoing positive selection. Likewise, both FUBAR and SLAC concurred on a common site 111 to be under positive selection. In contrast, FUBAR identified four negatively selected sites (73, 80, 87, and 145), while SLAC identified two sites (73 and 87) undergoing strong negative selection. Notably, both methods agreed on amino acid positions 73 and 87 experiencing negative selection. A plot depicting positions for positive and negative selection of the *PkCelTOS* protein sequence generated using SLAC is shown in Supplementary Figure S5.

### 3.6. B Cell Epitopes in PkCelTOS

The prediction of potential B-cell epitopes using the IEDB server in *P. knowlesi* CelTOS identified two surface-exposed epitopes (96AQLKATA102 and 124TIKPPRIKED133) towards the C’ terminal region of the protein (Table 3, Supplementary Figures S6 and S7). The Bcpred server predicted a single potential epitope (125IKPPRIKED133). Both the servers predicted a common region of nine amino acids (IKPPRIKED), which can serve as a potential region for vaccine development research, while upon analysis of epitopes with its closest ortholog, *P. vivax* CelTOS. Three epitope regions were identified by the IEDB server and four by the Bcpred Server (Supplementary Table S2). A comparative analysis of the epitopes between the two species identified a conserved region of four amino acids (KPPR) in length.

## 4. Discussion

The life-cycle of *Plasmodium* spp. involves alternating between a definitive invertebrate host and intermediate human hosts. When a mosquito takes a blood meal, the parasite is released in sporozoite form into the host skin. It then initiates gliding motility and cell traversal for penetrating different cell types, e.g., endothelial cells of blood vessels, dermal fibroblasts, phagocytes in sinusoidal and dermal layers (Kupffer cells), and hepatocytes [6,31,51,52]. This enables the parasites to surpass cellular barriers and access the hepatocytes, where they occupy and mature into liver-stage parasites. [6,31,51,52]. Cell traversal plays a crucial role in protecting sporozoites from destruction by phagocytes and preventing their arrest by nonphagocytic cells in the host dermis [53]. Thus, a vaccine against these sporozoite proteins can inhibit parasite transmission in the pre-erythrocytic stage. To ensure effectiveness across different geographical regions and to prevent an allele-specific immune response, it is preferable for a candidate antigen to exhibit minimal polymorphism [31]. CelTOS is one such sporozoite protein, well studied as a potential candidate for vaccine development against *P. vivax* and *P. falciparum*, but it has not yet been explored in *Plasmodium knowlesi*. The present study is a pioneer in investigating the genetic diversity and polymorphisms within the *PkCelTOS* gene in clinical samples from Malaysia, as well as predicting the B-cell epitopes that can be further studied to develop sporozoite vaccines.

By aligning 34 full-length amino acid sequences of *PkCelTOS*, it was observed that it exhibits the highest sequence identity with PvCelTOS, which may indicate the ongoing adaptation for invading the physical barriers of the human host’s immune system. The signal peptide region was conserved among all clinical samples of *P.knowlesi*, indicating that it may play a critical role in directing the newly synthesized protein to its intended destination, i.e., sporozoites and ookinetes. The results of a phylogenetic analysis by ML method did not show any geographical clustering as both the clusters had laboratory lines originating from Peninsular Malaysia and the Philippines, together with clinical samples from Sarawak, Malaysian Borneo. Similar results were also found by Assefa et al., 2015 using the same set of sequences [18], except that the laboratory strains formed a separate cluster from the clinical samples. However, many previous studies on vaccine candidates, e.g., Pk41 [29], PkRhopH2 [11], MSP4 [25], MSP1P [54], nbpxa [55] along with *P. knowlesi* genome sequences from clinical samples [56] has shown geographical clustering. The reason could be that *PkCelTOS* is expressed in sporozoite and ookinete surfaces and may not be exposed to human immune pressure. *PkCelTOS* was found to be phylogenetically most related to PvCelTOS, its ortholog infecting humans. Among the simian malaria parasites, *P. coatneyi* CelTOS is closer to *PkCelTOS.* The overall nucleotide diversity of *PkCelTOS* (π = 0.02111 ± 0.00105) was higher than the nucleotide diversity of *PfCelTOS* (π = 0.01001 ± 0.00036) worldwide samples [32] and 20 times higher than *PvCelTOS* (π = 0.00141 ± 0.00014) [33]. The nucleotide diversity of *PkCelTOS* was also found to be higher than other sporozoite stage vaccine candidates, e.g., PkTRAP (π = 0.00908 ± 0.0006) [17] and merozoite stage vaccine candidates like PkTRAMP (π = 0.00652 ± 0.00028) [10], PkRhopH2 (π = 0.00936 ± 0.0013) [11], Pk41 (0.00959 ± 0.0001) [29] but lower than PkMSP7D (π = 0.052+ 0.002) [12]. The low number of non-synonymous mutations than synonymous mutations may again attribute to the parasite adaptation in the new host.

Natural selection tests (using Tajima’s D, Fu and Li’s F* and D*) yielded positive results, implying that the *PkCelTOS* gene may be under positive selection; however, the results were statistically not significant, which may be due to a smaller sample size. However, dN-dS results are found to be negative but not significant. A higher number of non-synonymous mutations than synonymous mutations was found in the McDonald and Kreitman (MK) neutrality test between *PkCelTOS* orthologs. Codon-based site-by-site selection analyses in Datamonkey identified five potential sites which could be under positive natural selection and four sites under purifying selection. This is probably due to protein folding, and the sites under positive selection might be exposed to host immune pressure, while the negatively selected sites are under functional constraints and not exposed to the host. These findings may indicate that the gene in question is undergoing long-term differential selective pressure from the human immune system, similar to the conclusions of *PfCelTOS* [32]. Both IEDB and Bcpred servers predicted a four amino acid epitope region, which may be a potential candidate for cross-species vaccine development against *P. knowlesi* and *P. vivax*. Nevertheless, further sequence analysis involving a more extensive sample size would be necessary to substantiate the presence of the epitope.

## 5. Conclusions

To conclude, this present study provides a valuable understanding of the genetic diversity and polymorphism across the full-length gene of *CelTOS* gene in *Plasmodium knowlesi*, a malaria parasite prevalent in Southeast Asia. The high degree of nucleotide diversity observed in the *PkCelTOS* gene suggests this protein may be under selective host immune pressure. However, the identification of two potential epitopes on the *PkCelTOS* protein provides a promising avenue for the development of a vaccine that can stimulate an effective immune response. The phylogenetic analysis of *PkCelTOS* revealed two distinct groups, indicating that there may be genetic differences between *Plasmodium* populations in different regions of Malaysia. The absence of geographic clustering suggests that the genetic variation observed in the *PkCelTOS* gene is likely the result of selective local pressures rather than geographic isolation. In conclusion, this study highlights the need for continued surveillance of *PkCelTOS* genetic diversity in different regions. The findings of this study offer valuable knowledge for the rational design of peptide-based vaccines against *P. knowlesi* malaria. However, further functional studies, as well as genetic studies with a higher number of samples, are needed to validate the effectiveness of a *PkCelTOS*-based vaccine.

## Figures and Tables

**Table 1:** Calculation of nucleotide diversity, haplotype diversity, and neutrality indices for the *PkCelTOS* gene.

Gene	No. Samples	SNPs	Syn	Non-Syn	No. Haplotype	Diversity ± SD		Taj D	Fu and Li’s F*	Fu and Li’s D*
						Haplotype	Nucleotide			
*PkCelTOS*	34	28	12	16	17	0.954 ± 0.016	0.02111 ± 0.00105	1.52705 *p* > 0.10	1.10034 *p* > 0.10	0.63460 *p* > 0.10

SNPs: Single nucleotide polymorphisms, Syn: Synonymous substitutions, Non-Syn: Non-synonymous substitutions, SD: Standard deviation.

**Table 2:** Results of MK tests performed using the full-length *CelTOS* sequence of *P. knowlesi* with its orthologs in *P. caotneyi*, *P. vivax,* and *P. cynomolgi*.

*CelTOS*	Polymorphic Changes Observed in *P. knowlesi*		Fixed Differences between Species						Neutrality Index		
			*Pk* vs. *Pco*		*Pk* vs. *Pcy*		*Pk* vs. *Pv*		*Pk* vs. *Pco*	*Pk* vs. *Pcy*	*Pk* vs. *Pv*
	Syn	Non-Syn	Syn	NonSyn	Syn	Non-Syn	Syn	Non-Syn			
Full length	12	16	15	22	20	32	21	20	0.909	0.909	1.400

Syn: Synonymous sites, Non-Syn: Non-synonymous sites, *Pk*: Plasmodium knowlesi, *Pco*: Plasmodium coatneyi, *Pcy*: Plasmodium cynomolgi, *Pv*: Plasmodium vivax.

**Table 3:** Potential epitopes predicted in *P. knowlesi* CelTOS (ERR985408) by the IEDB and Bcpred server.

IEDB Server					Bcpred Server			
No.	Start AA	End AA	Peptide	Length	Start AA	End AA	Peptide	Length
1	96	102	AQLKATA	7	125	133	IKPPRIKED	9
2	124	133	TIKPPRIKED	10	-	-	-	NA

Bold font indicates the common amino acids within the predicted epitope as identified by both servers. The conserved epitope between the two species is highlighted in red amino acids. AA: amino acid.

## Data Availability

*PkCelTOS* gene sequences were downloaded from the public database European Bioinformatics Institute (EBI) (https://www.ebi.ac.uk/ena/browser/home accessed on 2 January 2023).

## Supplementary Materials

The following supporting information can be downloaded at: https://www.mdpi.com/article/10.3390/tropicalmed8080380/s1, Figure S1: Signal Peptide, Figure S2: Sequence identity between CelTOS othologs, Figures S3–S4: Nucleotide and Amino acid Polymorphisms, Figure S5: SLAC site graph, Figures S6–S7: Possible Epitopes; Table S1: Study samples with geographical origin.
