# Supplementary material for: In Silico Analysis Reveals High Levels of Genetic Diversity of Plasmodium knowlesi Cell Traversal Protein for Ookinetes and Sporozoites (PkCelTOS) in Clinical Samples
Source: Trop Med Infect Dis. 2023 Jul 26;8(8):380. doi: 10.3390/tropicalmed8080380 (PMC10458480; doi:10.3390/tropicalmed8080380)
Supplement: Supplementary file 1 [file tropicalmed-08-00380-s001.zip › Table S1.pdf]

Table S1. *PkCelTOS* Study samples with their geographical origin

No.	Accession number	Geographical Location
1	H-strain (PKNH_1436200)	Peninsular malaysia
2	PKNOH_S140254200 Malayan strain	Peninsular malaysia
3	MR4 (SRR2225571)	Peninsular malaysia
4	Philippine strain (SRR2225573)	Philippines
5	ERR274222	Sarikei, Malaysian Borneo
6	ERR366425	Sarikei, Malaysian Borneo
7	ERR366426	Sarikei, Malaysian Borneo
8	ERR985374	Betong, Malaysian Borneo
9	ERR985377	Betong, Malaysian Borneo
10	ERR985378	Betong, Malaysian Borneo
11	ERR985379	Betong, Malaysian Borneo
12	ERR985380	Betong, Malaysian Borneo
13	ERR985381	Betong, Malaysian Borneo
14	ERR985382	Betong, Malaysian Borneo
15	ERR985383	Betong, Malaysian Borneo
16	ERR985384	Betong, Malaysian Borneo
17	ERR985410	Betong, Malaysian Borneo
18	ERR985411	Betong, Malaysian Borneo
19	ERR985385	Kapit, Malaysian Borneo
20	ERR985387	Kapit, Malaysian Borneo
21	ERR985388	Kapit, Malaysian Borneo
22	ERR985389	Kapit, Malaysian Borneo
23	ERR985390	Kapit, Malaysian Borneo
24	ERR985392	Kapit, Malaysian Borneo
25	ERR985393	Kapit, Malaysian Borneo
26	ERR985394	Kapit, Malaysian Borneo
27	ERR985395	Kapit, Malaysian Borneo
28	ERR985397	Kapit, Malaysian Borneo

29	ERR985404	Kapit, Malaysian Borneo
30	ERR985406	Kapit, Malaysian Borneo
31	ERR985407	Kapit, Malaysian Borneo
32	ERR985408	Kapit, Malaysian Borneo
33	ERR985416	Kapit, Malaysian Borneo
34	ERR985418	Kapit, Malaysian Borneo
