# Supplementary figures and images for: In Silico Analysis Reveals High Levels of Genetic Diversity of Plasmodium knowlesi Cell Traversal Protein for Ookinetes and Sporozoites (PkCelTOS) in Clinical Samples
Source: Trop Med Infect Dis. 2023 Jul 26;8(8):380. doi: 10.3390/tropicalmed8080380 (PMC10458480; doi:10.3390/tropicalmed8080380)

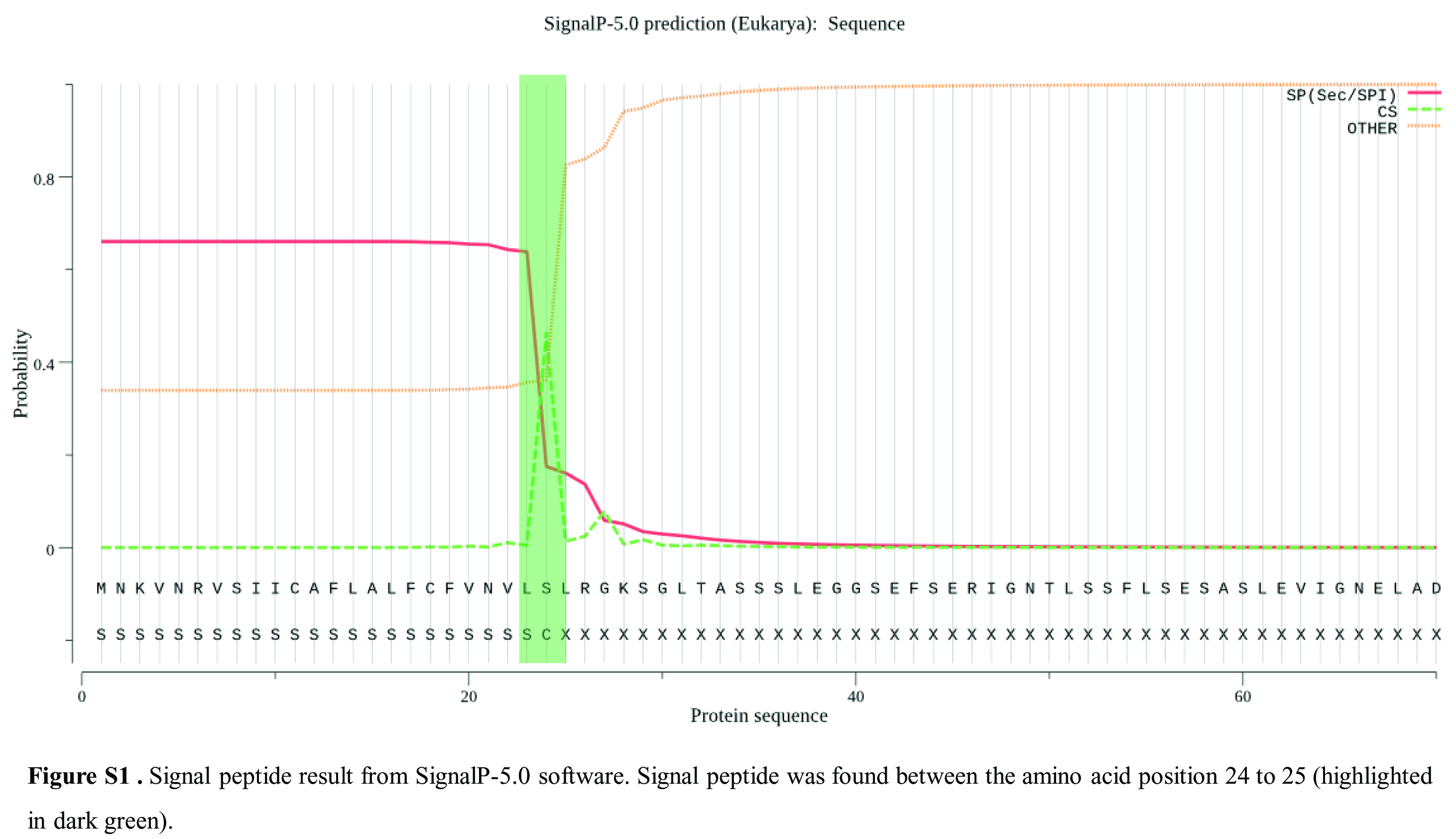

Supplement: Supplementary file 1 [file tropicalmed-08-00380-s001.zip › Suppl Fig S1.tif]

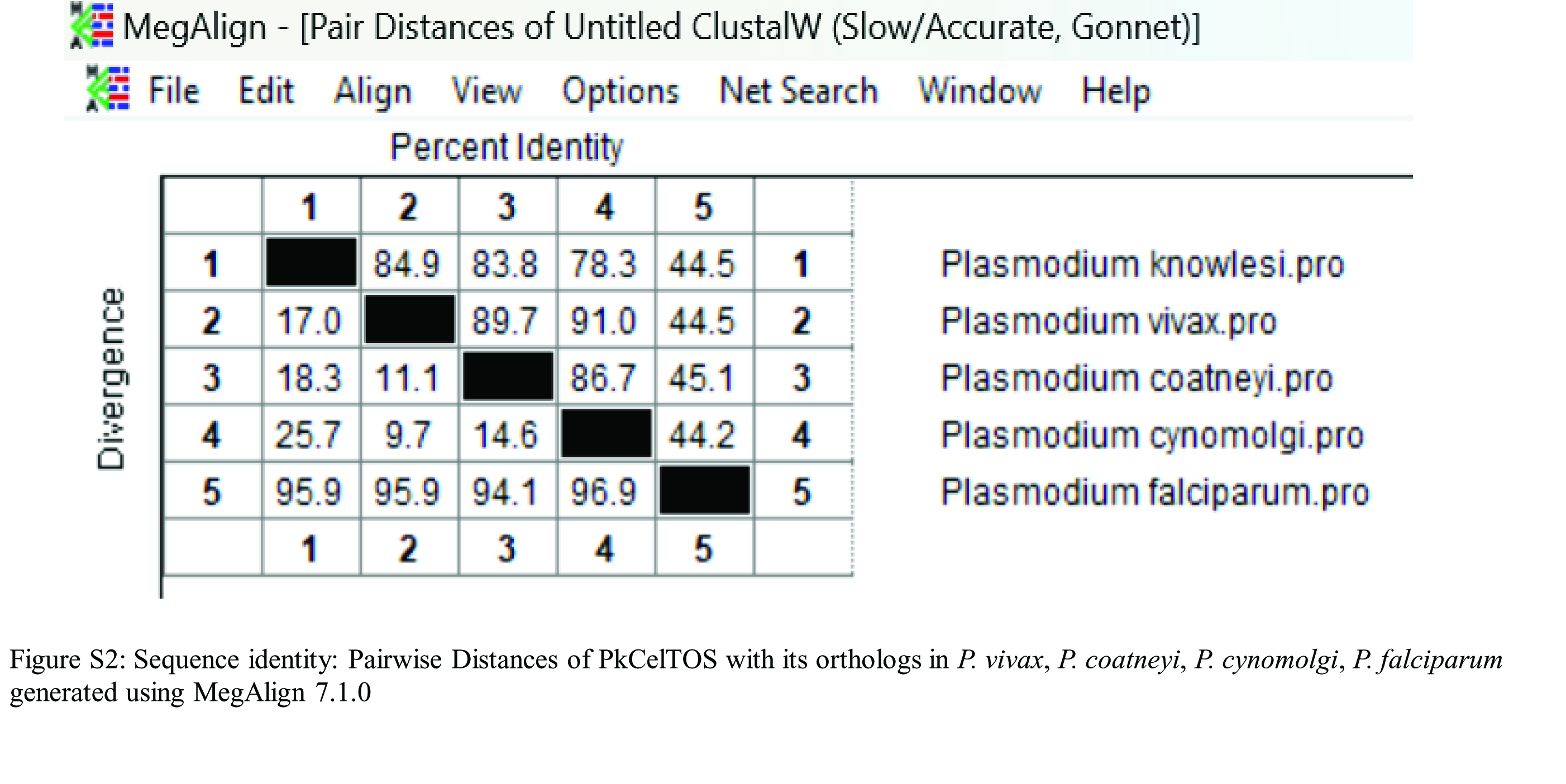

Supplement: Supplementary file 1 [file tropicalmed-08-00380-s001.zip › Suppl Fig S2.tif]

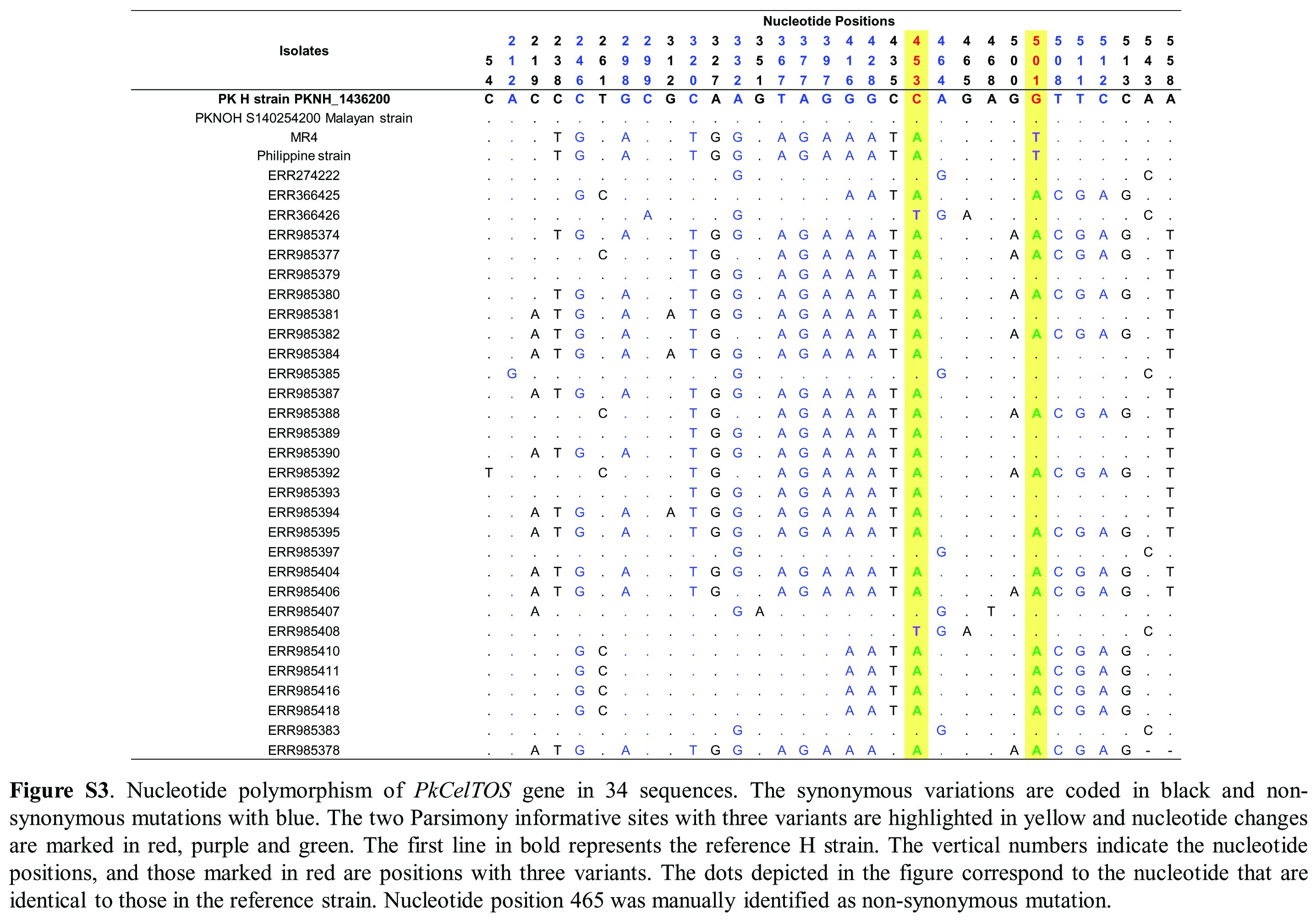

Supplement: Supplementary file 1 [file tropicalmed-08-00380-s001.zip › Suppl Fig S3.tif]

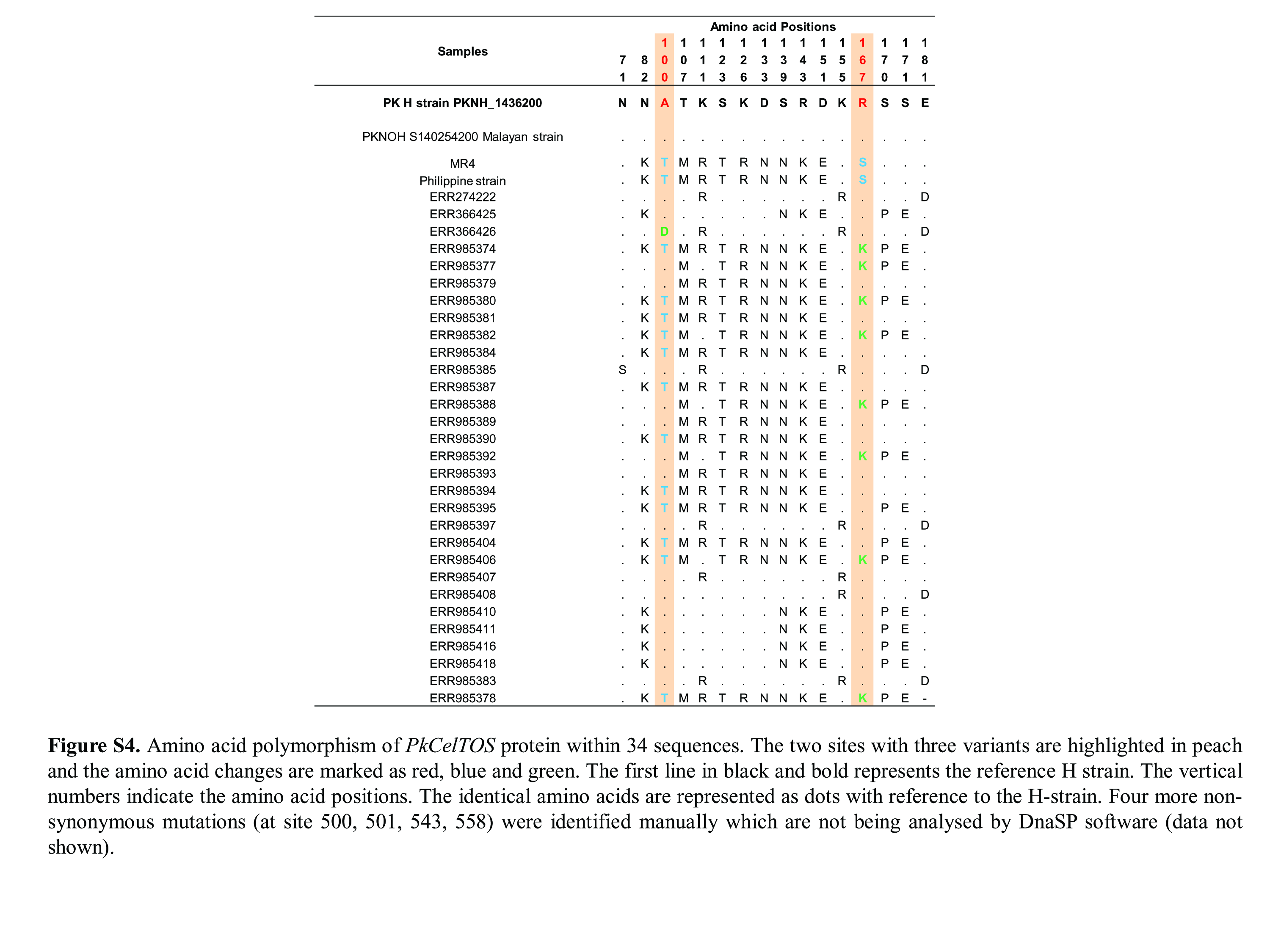

Supplement: Supplementary file 1 [file tropicalmed-08-00380-s001.zip › Suppl Fig S4.tif]

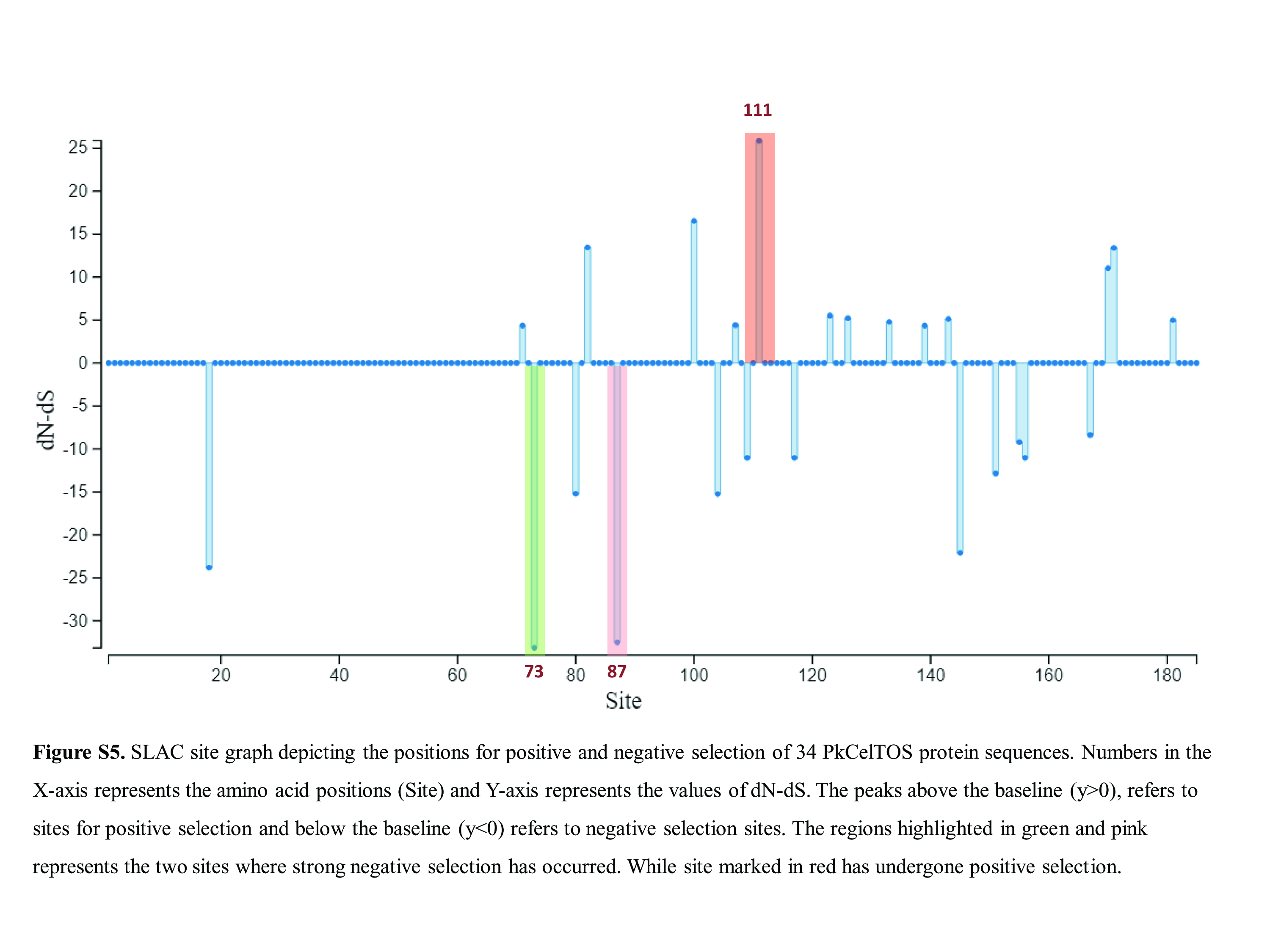

Supplement: Supplementary file 1 [file tropicalmed-08-00380-s001.zip › Suppl Fig S5.tif]

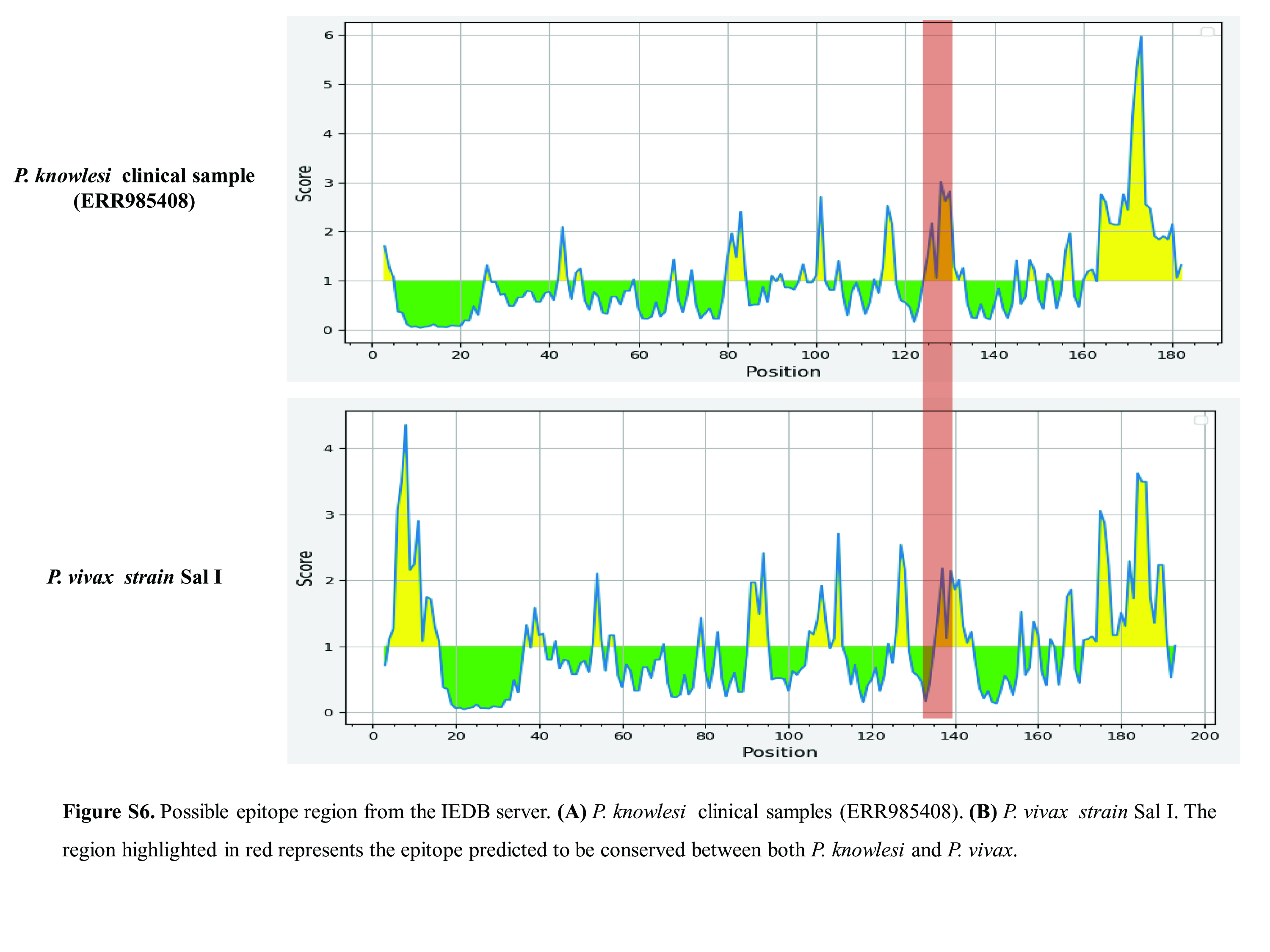

Supplement: Supplementary file 1 [file tropicalmed-08-00380-s001.zip › Suppl Fig S6.tif]

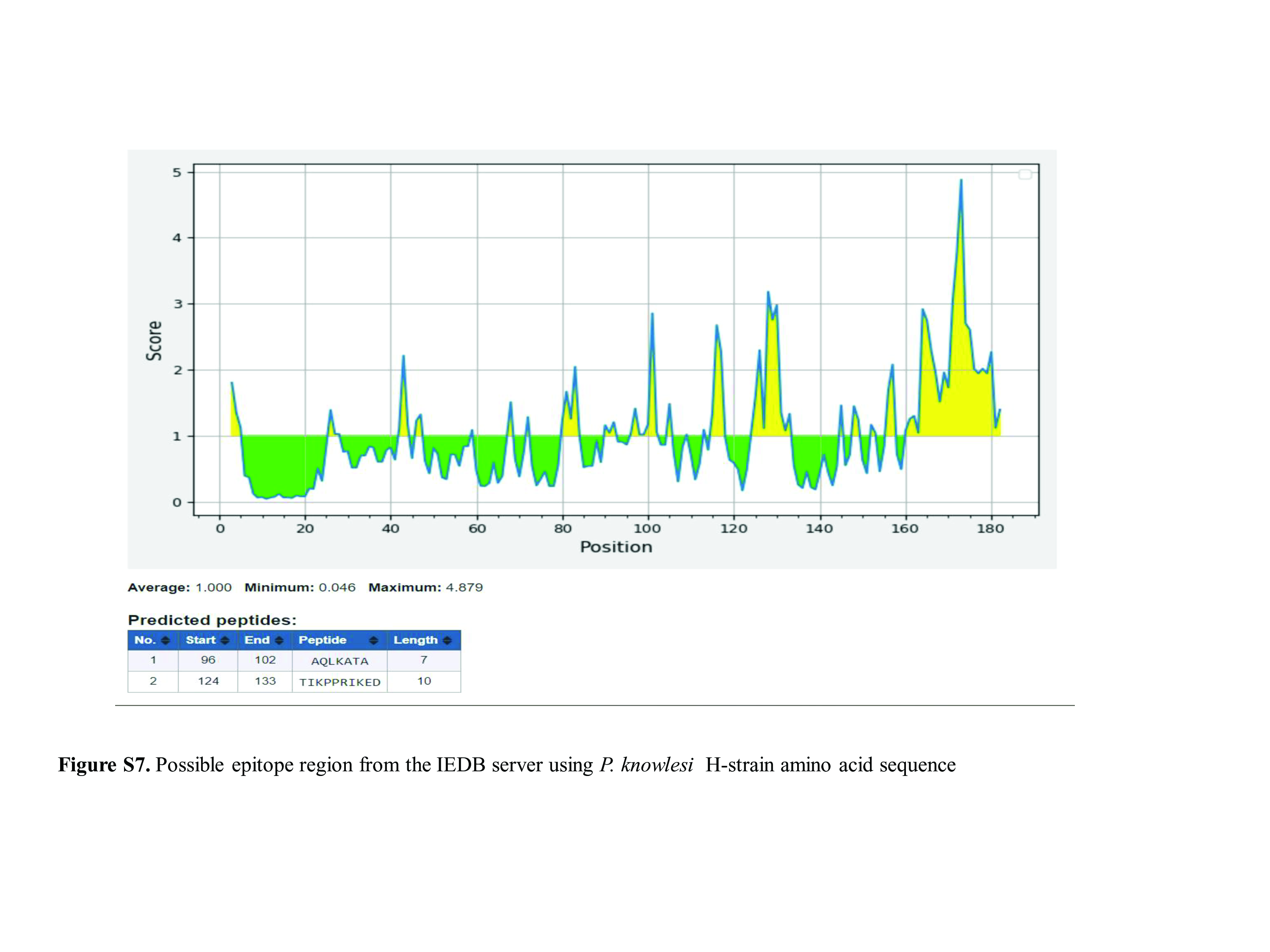

Supplement: Supplementary file 1 [file tropicalmed-08-00380-s001.zip › Suppl Fig S7.tif]
